# Effect of various types of extracellular DNA on *V. hyugaensis* biofilm formation

**DOI:** 10.1128/msphere.00035-23

**Published:** 2023-06-30

**Authors:** Carmen Gu Liu, Anthony W. Maresso

**Affiliations:** 1 Department of Molecular Virology and Microbiology, Baylor College of Medicine, Houston, Texas, USA; 2 TAILΦR: Tailored Antibacterials and Innovative Laboratories for Phage (Φ) Research, Baylor College of Medicine, Houston, Texas, USA; University of Michigan, Ann Arbor, Michigan, USA

**Keywords:** biofilm, extracellular DNA, biofilm formation, microscopy, *Vibrio*

## Abstract

Marine bacteria face a constant influx of new extracellular DNA (exDNA) due to the massive viral lysis that occurs in the ocean on a daily basis. Generally, biofilms have shown to be induced by self-secreted exDNA. However, the effect of various types of exDNA with varying lengths, self vs non-self, as well as guanine-cytosine content (GC) content on biofilm formation has not been explored, despite being a critical component of the extracellular polymeric substance. To test the effect of such exDNA on biofilms, a marine bioluminescent bacterium (*Vibrio hyugaensis*) was isolated from the Sippewissett Salt Marsh, USA, and treated with various types of exDNA. We observed rapid pellicle formation with distinct morphologies only in cultures treated with herring sperm gDNA, another *Vibrio* spp. gDNA, and an oligomer of 61–80% GC content. With pH measurements before and after the treatment, we observed a positive correlation between biofilm formation and the change to a more neutral pH. Our study highlights the importance of studying DNA-biofilm interaction by carefully examining the physical properties of the DNA and by varying its content, length, and source. Our observation may serve as the basis for future studies that seek to interrogate the molecular explanation for the various types of exDNA and their effects on biofilm formation.

**IMPORTANCE:**

Bacteria mostly exist as biofilm, a protective niche that promotes protection from the environment and nutrient uptake. By forming these structures, bacteria have caused recalcitrant antibiotic-resistant infections, contamination of dairy and seafood, and fouling equipment in the industry. A critical component that makes up the extracellular polymeric substances, the structural component of a biofilm, is the extracellular DNA secreted by the bacteria found in the biofilm. However, previous studies on DNA and biofilm formation have neglected the unique properties of nucleic acid and its high diversity. Our study aims at disentangling these DNA properties by monitoring their effect at inducing biofilm formation. By varying length, self vs non-self, and GC percentage, we used various microscopy techniques to visualize the structural composition of a *Vibrio hyugaensis* biofilm. We observed DNA-dependent biofilm stimulation in this organism, a novel function of DNA in biofilm biology.

## OBSERVATION

Extracellular DNA (exDNA) is found in many environments, with recent evidence suggesting its role in the human body, soil, marine life, and biofilms (1). In contrast to only being a passive product of lysed cells, they are found to be actively secreted by some cells for various tasks (1). For instance, they function in a neutrophil’s trap to immobilize pathogens in the human body, as a cargo for the transfer of genetic information across various environments, and/or as structural support for bacterial biofilms (1). ExDNA is usually depicted as a single, linear, and short molecule that affects various aspects of the biofilm. However, DNA is very diverse in terms of length (i.e., genomic DNA, plasmids, oligonucleotides, and single nucleotides), structure (i.e., A-DNA, B-DNA, C-DNA, D-DNA, and Z-DNA), content (i.e., individual genes and transcription factors), host and environmental source (i.e., bacteria, archaea, eukaryote, soil, marine, wastewater, etc.), and associated molecules (i.e., binding proteins such as histones, RNAs, and divalent cations). In the ocean, it is estimated that viruses lyse 20% of the marine microbial biomass, releasing a massive amount of exDNA on a daily basis (2). This in turn impacts the amount, diversity, and source of the exDNA that affects the microbiome within, especially their biofilm formation.

**Fig 1:**
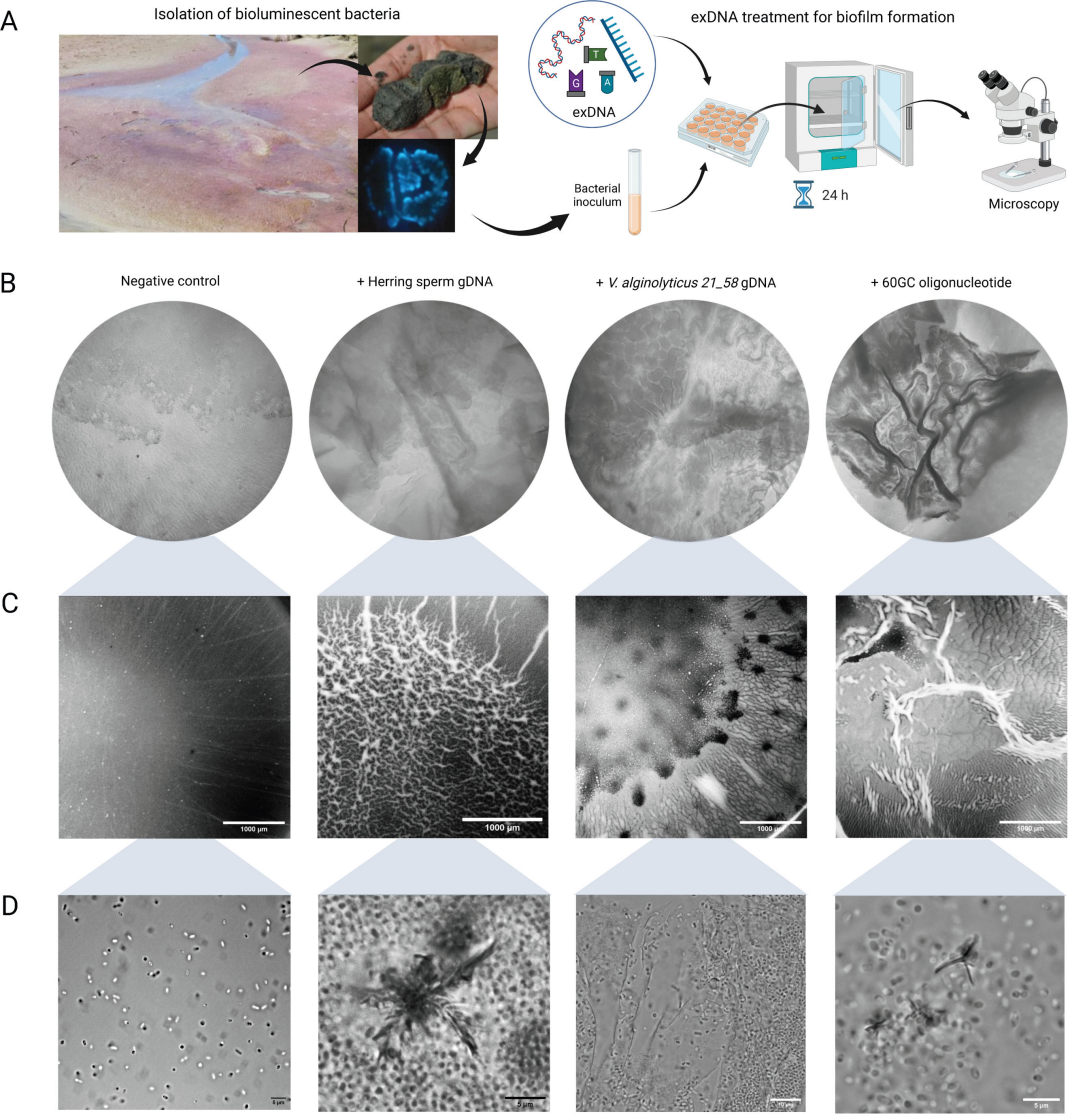
Pellicle biofilms formed with various DNA treatments at 24 h. (A) A diagram showing the isolation of a bioluminescent marine bacterium (*V. hyugaensis* CGL-A) from the Sippewissett Salt Marsh, USA in a seawater complete media. *V. hyugaensis* CGL-A was treated with various sources of exDNA, cultured statically for 24 h at 30°C, and assessed with various microscopes: (B) stereo microscope Zeiss AE2000, (C) dissecting microscope Zeiss Discovery V20, and (D) brightfield microscope Zeiss Imager M2. Three biological replicates were performed, and only representative images are shown.

Given that marine bacteria survive various environmental conditions by forming biofilms, it is especially important to understand the effect of various types of exDNA on biofilm formation. To do this, we isolated two bioluminescent bacteria, strain CGL-A and strain CGL-B, from a sediment found in the purple stream at the Sippewissett Salt Marsh, USA (Fig. 1A). Both isolates were streaked and purified for a total of five times in seawater complete (SWC) media grown at 30°C. We chose this marine bacterium because it forms a type of floating biofilm (i.e., pellicle biofilm) at the air-liquid interface that can be directly observed using various microscopes. To identify potential contaminants in the final isolated culture (strain CGL-A), gDNA was amplified using 515F and 806R primers targeting the V4 region for 16S amplicon sequencing (Illumina). Genomic sequences identified strain CGL-A as a pure, single bacterium similar to *Vibrio hyugaensis* (99.6% coverage and 98.8% identities) that was first reported in 2015 as a novel bacterium in Japan (3).

We hypothesize that different types of exDNA will induce different types of biofilms. To test this, exDNA from various sources was employed (Table 1). Based on this reasoning, we considered the effect of the size of DNA on biofilm induction. As such, we grouped the DNA into three main categories: (i) “genomic,” which is mostly composed of long strands of DNA of high molecular weight, (ii) “oligonucleotides,” which are short and of 20-mers, and finally, (iii) single “nucleotides” (Table 1). Within the genomic DNA, we incorporated a marine, non-bacterial eukaryotic source (herring sperm gDNA, non-self, abbreviated as “HS”), a related *Vibrio* species (*V. alginolyticus 21_58,* non-self, abbreviated as “VD”), the same species isolated from the same source (*V. hyugaensis CGL-B*, non-self, abbreviated as “VB”), and the very own genomic DNA of the testing strain (*V. hyugaensis* CGL-A, self, abbreviated as “VA”). As for the oligonucleotides, these are short DNA strands that do not encode genes, as opposed to genomic DNA. In short, we tested the effect of various oligomers that differ on their GC content percentage: 0–20%, 21–40%, 41–60%, 61–80%, and 81–100%. Finally, we incorporated the possible effect of all four single nucleotides: dATP, dCTP, dGTP, and dTTP (Table 1). As opposed to the previous two categories, nucleotides are small organic molecules devoid of genes and complex three-dimensional structures.

**TABLE 1:** Different DNA samples for biofilm induction, their acronyms, final concentration, and source

Category	DNA sample	Acronym	Final concentration	Source
Genomic	Herring sperm	HS	1 ng/µL	Promega (catalog no. D1811)
	*V. hyugaensis* CGL-A	VA	1 ng/µL	This study
	*V. hyugaensis* CGL-B	VB	1 ng/µL	This study
	*V. alginolyticus* 21_58	VD	1 ng/µL	Marine Biological Laboratory
Oligonucleotides	0–20% GC	0GC	0.1 µM	IDT (catalog no. 51-01-15-08)
	21–40% GC	20GC	0.1 µM	IDT (catalog no. 51-01-20-01)
	41–60% GC	40GC	0.1 µM	IDT (catalog no. 51-01-19-06)
	61–80% GC	60GC	0.1 µM	IDT (catalog no. 51-01-18-02)
	81–100% GC	80GC	0.1 µM	IDT (custom: GGCCGGCCGGCCGGCC)
Nucleotides	dATP	dATP	1 µM	Promega (catalog no. U1410)
	dCTP	dCTP	1 µM	
	dGTP	dGTP	1 µM	
	dTTP	dTTP	1 µM	

To make sure that overnight incubation does not release substantial exDNA that may interfere with our assay, we measured exDNA produced by the overnight cultures themselves (strains CGL-A and CGL-B). After centrifuging the overnight cultures, the supernatant of each strain was recovered into a fresh microcentrifuge tube, and the concentration of the DNA was measured using the Quantus Fluorometer system. Overall, the levels of self-exDNA in the overnight cultures were non-detectable, which suggests minimal interference from self-released exDNA.

*V. hyugaensis* CGL-A was treated with various sources of exDNA (Table 1) for 24 h with static growth that allows uninterrupted formation of the pellicle biofilm, followed by assessment via various microscopy techniques (inverted, brightfield, stereo, and transmission electron). Interestingly, the treatment of *V. hyugaensis* CGL-A with the HS, VD, and a single-stranded 20-mer DNA of the 61–80% GC category (abbreviated as “60GC”; non-self, oligomer) resulted in very distinct pellicle biofilms, which were absent in the rest of the DNA-treated cultures (Fig. 1B and C; see acronyms in Table 1). To note, we did not observe noticeable biofilm in VA- (self) and VB-treated (same source) cultures. To confirm that exDNA from a non-self-source effects biofilm formation, we assessed untreated vs HS-treated cultures via transmission electron microscopy (TEM) and noticed a widespread sheet-like structure surrounding individual bacterium (Fig. S1). When the pellicle biofilm was sampled for brightfield microscopy (Fig. 1D), we noticed the presence of rosette-like structures in both HS- and 60GC-treated cultures. When these were stained with DAPI (for DNA), we confirmed that the rosette-like structures were not DNA molecules (data not shown). In contrast, the VD-treated samples have a completely different structure: instead of the rosettes, they have extensive filaments that together form a sheet-like structure (Fig. 1D). When this sample was treated with DNAse I for an hour, the sheet-like structure was not visible. Instead, loosely attached long filaments of bacteria were evident, which suggests that the long filaments are composed of DNA wrapping individual cells together (Fig. S2). Collectively, these observations indicate that our isolated *V. hyugaensis* CGL-A has unique preferential reaction toward the type of exDNA for pellicle biofilm formation and that it induces different morphological changes (both macro- and microscopical) based on the exDNA treated.

**Fig 2:**
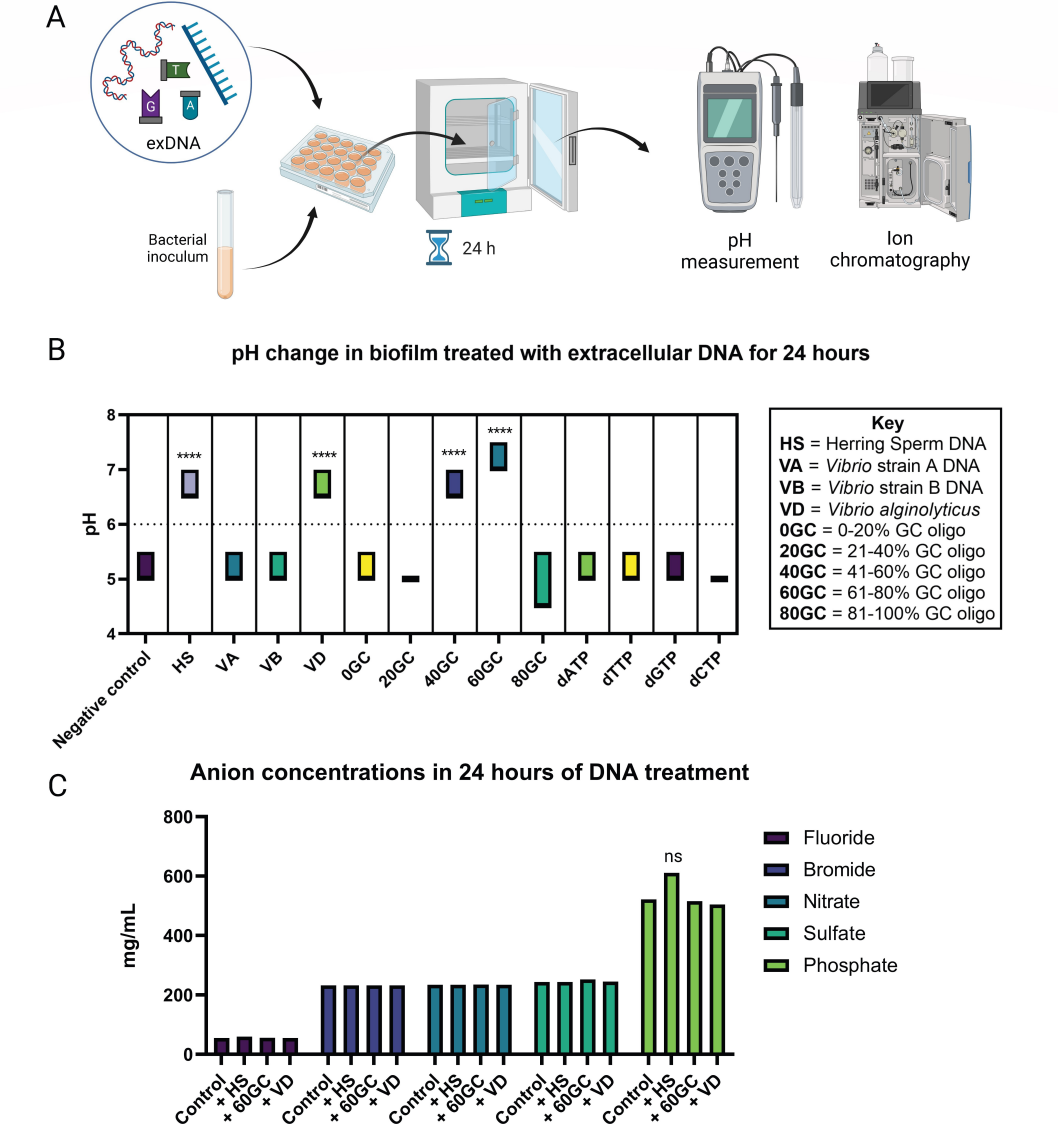
Change of pH and anion concentrations in biofilms treated with various sources of exDNA for 24 h. (A) A diagram showing the treatment of exDNA on *V. hyugaensis* CGL-A in a 12-well plate, grown for 24 h statically at 30°C. Cultures were then tested for (B) pH change in the biofilm as well as (C) anion concentrations. The dashed line represents pH measurements at *t* = 0 h. For statistical significance testing, one-way ANOVA was employed for the pH testing, and two-way ANOVA was employed for the anion testing. *, *P* < 0.05; **, *P* < 0.01; ***, *P* < 0.001; ns, not significant.

Finally, since the addition of exDNA has been shown to acidify a biofilm (4), we measured the pH in all exDNA-treated cultures before and after the treatment. It turns out that HS-, VD-, 40GC-, and 60GC-treated samples were more neutral than the starting pH (~6.0), while the rest were more acidic (pH ~5) (Fig. 2B). This is positively correlated with the distinct pellicle biofilm phenotypes observed through various microscopy techniques in these exDNA-treated samples, except the 40GC-treated culture. Our data correlate with previous studies that have shown more biofilm growth at a more neutral pH (5, 6). Finally, to test whether the added DNA is being used as a phosphorus source like *Shewanella* (7), a panel of anion concentrations was measured (Fig. 2C). Phosphorus level in DNA-treated samples was not statistically different from the control, suggesting that DNA is not being used as a phosphorus source and thus not promoting biofilm formation as a nutrient.

Biofilms have many implications in ecology, microbial biology, bioremediation, wastewater treatment, and medicine (8 - 10). It affects how bacteria socialize, colonize, aggregate, and survive in various environments as a collective biological unit. As such, biofilm formation heavily depends on the available nutrients and substrates that bacteria can utilize in each microenvironment. In this study, we sought to test whether certain types of DNA (gDNA, oligonucleotides, single nucleotides, self, and non-self) can induce a response from bacteria and whether they can lead to forming different types of biofilms. In summary, we observed that (i) various types of exDNA induce different biofilm formations in *V. hyugaensis* and that (ii) this is positively correlated with the change to a more neutral pH. Our isolated bacterium responds to two non-self genomic DNA (herring sperm and another *Vibrio* spp.), as well as the oligonucleotide of 20-mer which contains between 61% and 80% GC content.

The nature of DNA is acidic due to the phosphorus group, and the addition of exDNA has shown to acidify bacterial planktonic as well as biofilm cultures (4). Instead, we observed that the addition of some exDNA is correlated to a more neutral pH in biofilm-positive cultures. Since a recent study found that exDNA controls gene expression (especially genes involved in nutrient utilization, acid pH tolerance, and virulence) in *Pseudomonas aeruginosa* (11), it could be that the addition of those specific exDNA has altered gene expression in this isolate. It may have not only allowed survival at acidic pH, but it may have altered the pH as a response to those specific exDNA, which in turn have allowed more growth and biofilm formation. It would be interesting for future studies to investigate whether altering gene expression with exDNA can also directly alter biofilm formation. Finally, since exDNA is a structural biofilm component, we believe that even without altering gene expression, DNA by itself can shape biofilm formation.

Overall, this study underscores the diversity of exDNA and its impact on biofilm formation, which can be relevant to species that form heavy biofilms in the presence of exDNA. Future studies may include investigating the various DNA preferences (with varying structures and length) by each bacterium and whether there is a self-recognition at the nucleic acid level when these are present extracellularly (in contrast to the CRISPR system, which recognizes intracellular DNA).

## EXPERIMENTAL PROCEDURES

### Bacterial isolation and growth conditions

Two bioluminescent bacteria, strain CGL-A (sample ID #22.4_1.CGL.011) and strain CGL-B (sample ID #22.4_1.CGL.012), were isolated from a sediment found in the purple stream at the Sippewissett Salt Marsh (sample ID #22.CGL.9.7.4), USA. Both isolates were streaked for a total of five times for isolation and stored at −80°C. All isolates were grown in SWC (5 g of tryptone, 1 g of yeast extract, 3 mL of glycerol, 5 mL of 1 M MOPS, pH 7.2 in 1 L of 1× seawater base) media at 30°C. The seawater base media are composed of final 423 mM NaCl, 22.9 mM MgCl_2_·6H_2_O, 9.2 mM CaCl_2_·2H_2_O, and 9.0 mM KCl. All recipes are derived from the Marine Biological Laboratory, Woods Hole, MA, USA through the Microbial Diversity Course (12).

### DNA isolation

To isolate large amounts of DNA, 500 mL of overnight cultures was centrifuged (5,000 rpm, 15 min, 4°C), and pellets were resuspended with equal volumes of the lysis buffer from the PureYield Plasmid Maxiprep Kit (Promega, catalog no. A2392). This resuspension was centrifuged (5,000 rpm, 15 min, 4°C), and the supernatant was recovered. Then, 10 µL of 3 M sodium acetate per milliliter of supernatant was added, followed by adding an equal volume of isopropanol and incubating for 2 h at −20°C (13). Pellet was recovered after centrifugation (5,000 rpm, 15 min, 4°C), washed with two rounds of 70% ice-cold ethanol, and resuspended in nuclease-free H_2_O (13). All DNA quantities were determined with the Quantifluor ONE dsDNA System (Promega, catalog no. E4870) using Quantus Fluorometer (Promega, catalog no. E6150) according to the manufacturer’s instructions (14).

### 16S amplicon sequencing and analysis

To identify potential contaminants in the final isolated culture (strain CGL-A), gDNA was amplified using 515F and 806R primers targeting the V4 region (15). Then, 16S amplicon sequencing (Illumina) was performed, resulting in 250 bp reads length (Accession: PRJNA983429). These reads were paired, trimmed, and merged, and sequences were extracted with a minimum length of 150 bp and a maximum length of 290 bp (15). These reads were clustered into operational taxonomic units (OTUs) using Geneious Prime 2023.0.4 (https://www.geneious.com) *de novo* assembler, resulting in consensus sequences and unused reads. To identify the organisms, both reads were BLASTn in the 16S microbial database.

### Secreted DNA assay

To measure exDNA produced by the cultures themselves, 1 mL of the overnight cultures (strains CGL-A and CGL-B) was removed and centrifuged (5,000 rpm, 15 min, 4°C). The supernatant of each strain was recovered into a fresh microcentrifuge tube, and the concentration of the DNA was measured using the Quantus Fluorometer system according to the manufacturer’s instructions (14).

### DNA treatment assay

To determine the effect of DNA on biofilm, strain CGL-A was grown overnight and diluted 1:1,000 into a 12-well plate containing 2 mL of SWC per well with various DNA treatments (see Table 1). Biofilm was grown for a total of 24 h, and both microscopy and pH measurement were performed. DNA-treated cultures were visualized with the stereo microscope Zeiss AE2000, dissecting microscope Zeiss Discovery V20, and brightfield/fluorescence microscope Zeiss Imager M2. One-way ANOVA testing was conducted as statistical analysis for the pH measurements.

### DNAse treatment assay

To determine whether DNA is responsible for the film-like structures on VD-biofilms, DNAse I (final 10 µg/mL) was applied to the 24-h biofilm for 1 h incubation at 30°C [adapted from reference (16)]. Immediately after incubation, biofilm structures were assessed with the Zeiss Imager.M2 microscope.

### Ion chromatography

To assess the anion concentrations of the biofilms, 1 mL of the 24 h biofilms (control, HS-treated, VD-treated, and 60GC-treated) was centrifuged (5,000 rpm, 15 min, 4°C), and the supernatant was recovered and diluted 1:100 into water for ion chromatography (Thermo Scientific Integrion HPIC with Dionex AS-AP) according to the manufacturer’s instructions (17). Two-way ANOVA was employed for statistical significance testing.

### Transmission electron microscopy

Biofilm (at 24 h) of the control (no treatment) and DNA-treated (HS) samples was positively stained with 2% uranyl acetate solution for 2 min, washed twice with MilliQ water, and imaged with TEM (18).

## SUPPLEMENTAL MATERIAL

Supplemental Material msphere.00035-23-s0001.docx Figure S1. Transmission electron microscopy (TEM) images of the negative control and the pellicle biofilm formed with herring sperm gDNA treatment. Figure S2. DNase I treatment of *V. alginolyticus* 21_58 biofilm.

ASM does not own the copyrights to Supplemental Material that may be linked to, or accessed through, an article. The authors have granted ASM a non-exclusive, world-wide license to publish the Supplemental Material files. Please contact the corresponding author directly for reuse.
