# Supplementary material for: Effect of various types of extracellular DNA on V. hyugaensis biofilm formation
Source: mSphere. 2023 Jun 30;8(4):e00035-23. doi: 10.1128/msphere.00035-23 (PMC10449505; doi:10.1128/msphere.00035-23)
Supplement: Supplemental Material — Figure S1. Transmission electron microscopy (TEM) images of the negative control and the pellicle biofilm formed with herring sperm gDNA treatment. Figure S2. DNase I treatment of V. alginolyticus 21_58 biofilm. [file msphere.00035-23-s0001.docx]

**Supplemental Materials for**

**EFFECT OF VARIOUS TYPES OF EXTRACELLULAR DNA ON *V. HYUGAENSIS* BIOFILM FORMATION**

**Carmen Gu Liu^12^ and Anthony W. Maresso^12^**

^1^ Department of Molecular Virology and Microbiology, Baylor College of Medicine, Houston, Texas, 77030, U.S.A.

^2^ TAILΦR: Tailored Antibacterials and Innovative Laboratories for phage (Φ) Research, Baylor College of Medicine, Houston, Texas, 77030, U.S.A.

**Correspondence: maresso@bcm.edu**

**Keywords: biofilm, extracellular DNA, biofilm formation, microscopy**


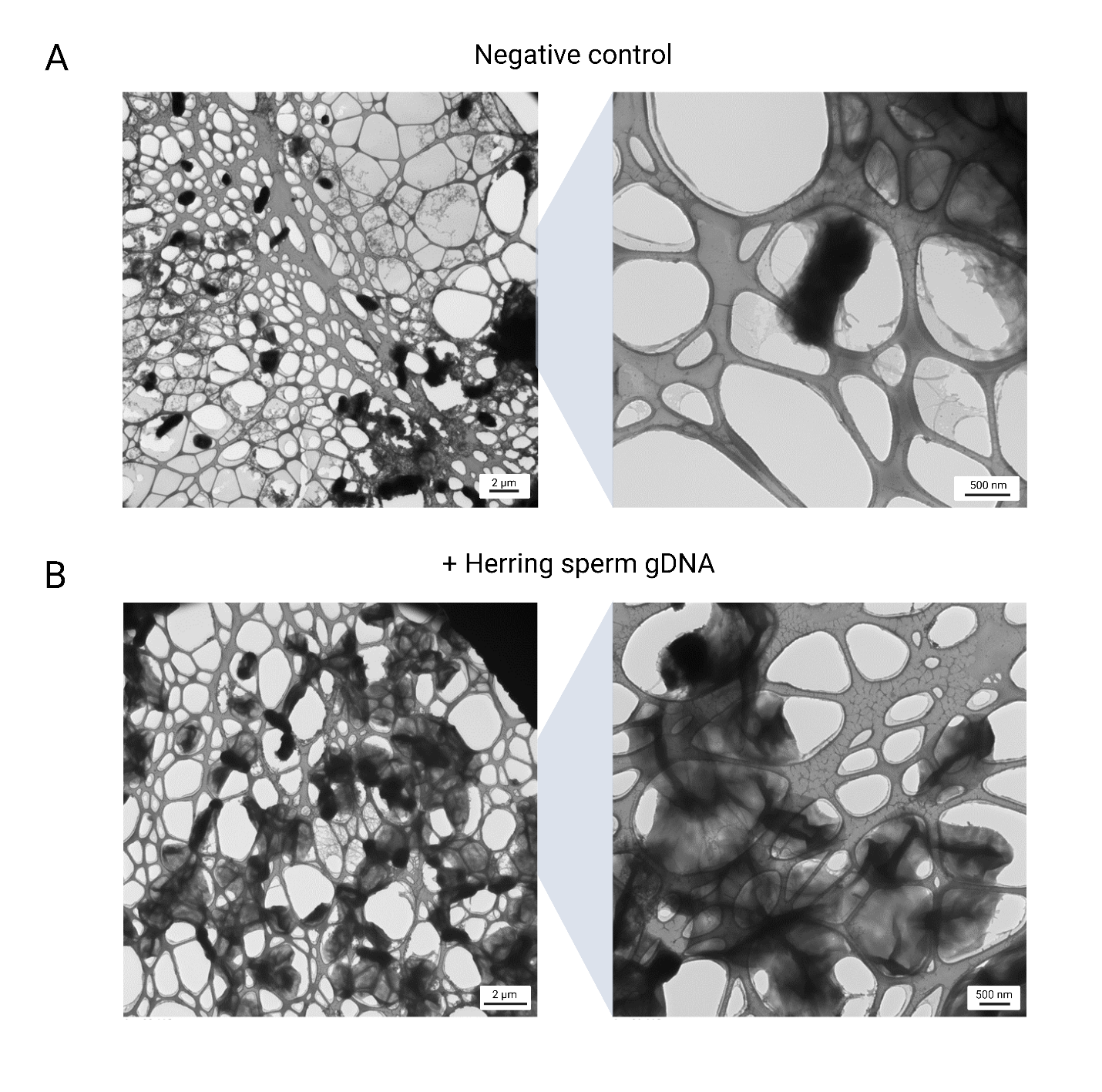


**Supplemental Figure 1. Transmission electron microscopy (TEM) images of the negative control and the pellicle biofilm formed with herring sperm gDNA treatment.** *V. hyugaensis CGL-A* was treated with herring sperm gDNA, cultured statically for 24 hours at 30°C and assessed with transmission electron microscopy. A) Negative control (untreated) and B) exDNA-treated samples were positively stained, washed, and imaged. The background mesh belongs to the TEM grid.


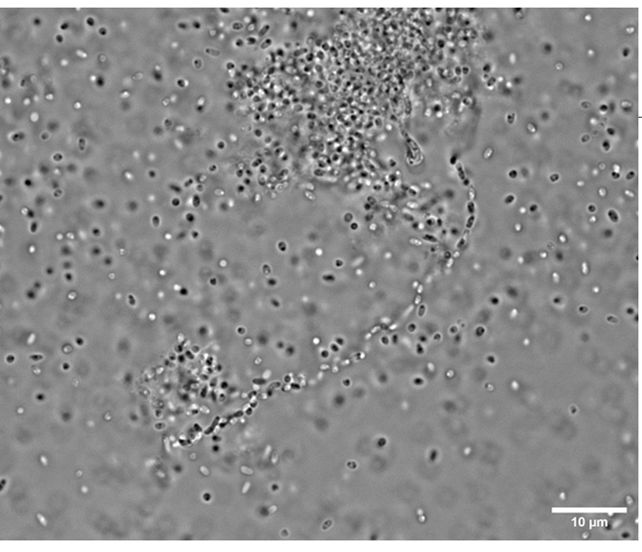


**Supplemental Figure 2. DNAse I treatment of** ***V. alginolyticus 21_58* biofilm.** A 24-hour biofilm of *V. alginolyticus 21_58* was treated with DNAse I (final 10 ug/mL) for one hour at 30°C and imaged with the Zeiss Imager.M2 microscope.
